# The Effect of Atorvastatin (and Subsequent Metformin) on Adipose Tissue Acylation-Stimulatory-Protein Concentration and Inflammatory Biomarkers in Overweight/Obese Women With Polycystic Ovary Syndrome

**DOI:** 10.3389/fendo.2019.00394

**Published:** 2019-06-25

**Authors:** Thozhukat Sathyapalan, James P. Hobkirk, Zeeshan Javed, Sean Carroll, Anne-Marie Coady, Philip Pemberton, Alexander Smith, Katherine Cianflone, Stephen L. Atkin

**Affiliations:** ^1^Department of Academic Diabetes, Endocrinology and Metabolism, Hull York Medical School, University of Hull, Kingston upon Hull, United Kingdom; ^2^Department of Sport, Health and Exercise Science, University of Hull, Kingston upon Hull, United Kingdom; ^3^Department of Obstetric Ultrasound, Hull and East Yorkshire Women's and Children's Hospital, Kingston upon Hull, United Kingdom; ^4^Specialist Assay Laboratories, Manchester Royal Infirmary, Manchester, United Kingdom; ^5^Centre de Recherche Institut Universitaire Cardiologie, Laval Université, Quebec City, QC, Canada; ^6^Weill Cornell Medical College Qatar, Education City, Doha, Qatar

**Keywords:** atorvastatin, acylation-stimulating-protein, interleukin-6, monocyte-chemoattractant-protein-1, adipose tissue, PCOS

## Abstract

**Background:** Atorvastatin has been shown to improve cardiovascular risk (CVR) indices in women with polycystic ovary syndrome (PCOS). Low-grade chronic inflammation of adipose tissue may link PCOS and adverse CVR. In pro-inflammatory states such as PCOS, spontaneous activation of the alternative pathway of complement results in increased generation of acylation stimulating protein (ASP) from adipocytes irrespective of body mass index.

**Methods:** The objective of this study was to determine the effect of atorvastatin on markers of adipose tissue dysfunction and inflammation; acylation-stimulating-protein (ASP), interleukin-6 (IL-6), and monocyte-chemoattractant-protein-1 (MCP-1) in PCOS. This was a randomized, double-blind, placebo-controlled study where 40 medication-naive women with PCOS and biochemical hyperandrogenaemia were randomized to either atorvastatin 20 mg daily or placebo for 12 weeks. Following the 12 week randomization; both group of women with PCOS were subsequently started on metformin 1,500 mg daily for further 12 weeks to assess whether pre-treatment with atorvastatin potentiates the effects of metformin on markers of adipose tissue function We conducted a *post-hoc* review to detect plasma ASP and the pro-inflammatory cytokines IL6 and MCP-1 before and after 12 and 24 weeks of treatment.

**Results:** There was significant reduction in ASP (156.7 ± 16.2 vs. 124.4 ± 14.8 ng/ml p <0.01), IL-6 (1.48 ± 0.29 vs.0.73 ± 0.34 pg/ml *p* = 0.01) and MCP-1 (30.4 ± 4.2 vs. 23.0 ± 4.5 pg/ml *p* = 0.02) after 12 weeks of atorvastatin that was maintained subsequently with 12 weeks treatment with metformin. There was a significant positive correlation between ASP levels with CRP (*p* < 0.01), testosterone (*p* < 0.01) and HOMA-IR (*p* < 0.01); IL-6 levels with CRP (p <0.01) and testosterone (*p* < 0.01) and MCP-1 with CRP (*p* < 0.01); testosterone (*p* < 0.01) and HOMA-IR (*p* < 0.02).

**Conclusions:** This *post-hoc* analysis revealed that 12 weeks of atorvastatin treatment significantly decreased the markers of adipose tissue dysfunction and inflammation, namely ASP, IL-6 and MCP-1 in obese women with PCOS. Changes in adipose tissue markers were significantly associative with substantial improvements in HOMA-IR, testosterone and hs-CRP levels.

**ISRCTN Number:** ISRCTN24474824.

## Introduction

In sub-clinical? pro-inflammatory states, including polycystic ovary syndrome (PCOS), spontaneous activation of the alternative pathway of complement results in increased generation of acylation stimulating protein (ASP) from adipocytes irrespective of body mass index (BMI) (1, 2). ASP is formed with the cleavage of Complement 3a,by carboxypeptidase B, that removes arginine at the carboxy-terminal generating C3adesArg (3). ASP contributes to storage of energy as lipids within adipose tissue by augmenting post-prandial triglyceride (TG) clearance, enhanced fatty acid esterification to triglycerides and decreased lipolysis through inhibiting hormone sensitive lipase (HSL), thereby increasing insulin resistance (2, 4, 5). ASP impacts fat storage by enhancing diacylglycerol acyltransferase (DGAT) activity, the rate limiting step in TG synthesis, stimulating glucose transporter GLUT4 translocation and indirectly enhancing LPL (lipoprotein lipase) activity in adipocytes (6, 7).

Moreover, ASP has been shown to be raised in other insulin resistant states, such as diabetes and obesity, as well as in cardiovascular disease and is reported to be positively correlated to plasma triglycerides, LDL cholesterol and non-esterified fatty acids (NEFA) all of which are common in women with PCOS (5, 8). It has been suggested that high levels of ASP found in PCOS represent an ASP-resistant state, that is related to “adipose tissue dysregulation” which subsequently leads to adipose tissue inflammation, insulin resistance, and dyslipidemia in these patients (9). The suggested possible underlying mechanism of ASP resistance in such patients is reduced expression of C5L2 receptor on adipose tissue (10, 11). Interestingly, this process of decreased C5L2 expression is enhanced further by increased fatty acids and high inflammatory markers present in obesity. Further, raised ASP levels itselfinduces pro-inflammatory state via increased cytokine release, as evident by both *in vitro* and *in vivo* studies (12, 13). The underlying molecular mechanisms of ASP resistance involve down regulation of intracellular response elements, especially AKT, PI3-kinase and PDK. These are also vital components of insulin response, which could link the development of insulin resistance in conditions such as PCOS (14).

It is also well-established that women with PCOS have underlying chronic low grade inflammation associated with high levels of inflammatory markers especially interleukin-6 (IL-6), macrophage chemoattractant protein-1 (MCP-1) and highly sensitive C-reactive protein (hs-CRP)—that are independent of obesity (15). Metformin has been shown to reduce markers of inflammation, especially ASP levels, in non-obese PCOS women (9, 16). We have shown previously that atorvastatin therapy reduced the inflammatory marker (hs-CRP) and insulin resistance, which were positively correlated with the reduction in triglyceride levels (17). We hypothesized that the effect of atorvastatin on measures of inflammation and insulin resistance are mediated through changes in ASP that have not been explored before in women with PCOS. Accordingly, this *post-hoc* analysis was performed to investigate the effects of atorvastatin and metformin on inflammatory markers, including ASP in a well-characterized group of young overweight/obese females with PCOS (17).

## Materials and Methods

The diagnosis of PCOS was based on all three diagnostic criteria of the Rotterdam consensus, namely clinical and biochemical evidence of hyperandrogenaemia (Ferriman-Gallwey score >8; free androgen index >8, respectively), oligomenorrhea or amenorrhea and polycystic ovaries on transvaginal ultrasound (18). Non-classical 21-hydroxylase deficiency, hyperprolactinaemia, Cushing's disease and androgen-secreting tumors were excluded by appropriate tests. All women gave their written informed consent and the study was approved by the South Humber Research Ethics committee, UK (REC reference: 04/Q1105/60).

**Figure 1:**
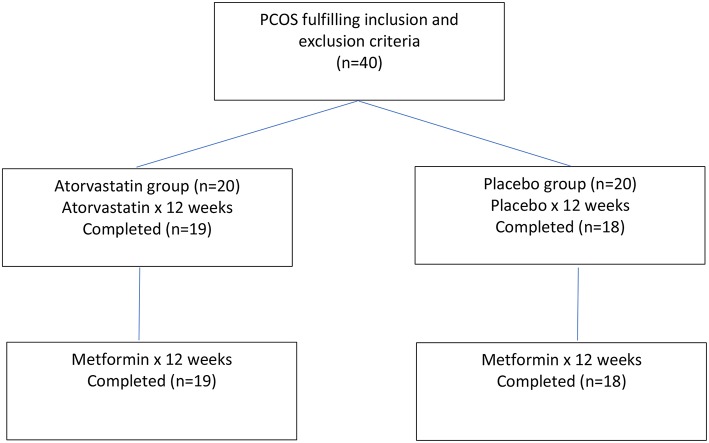
Flow chart of participants through the study.

Forty medication naïve women with PCOS and biochemical hyperandrogenaemia were randomized to atorvastatin 20 mg daily or placebo for 3 months (17). Following this, an extension study for both PCOS groups of women was undertaken with metformin 1,500 mg daily after completing the initial 3 months of atorvastatin or placebo (19). A Flow chart of participants through the experimental protocol is provided within Figure 1. The purpose of the extension study was to see whether pre-treatment with atorvastatin potentiates the effects of metformin on measures of inflammation and adipose tissue dysfunction

Thirty-seven women (atorvastatin = 19; placebo = 18) completed 6 months of this study. Compliance was monitored by counting returned medication. The biochemical assays undertaken were fully described in detail in previous publications of this investigation (17, 19). The same population is used in both this analysis and previous studies (17, 19).

ASP levels were measured in plasma C3 in plasma samples, in-house plasma controls and standard ASP were PEG precipitated (polyethylene glycol 8000, Fisher Scientific). The samples were centrifuged and the supernatant was used to assay for ASP. Plates were pre-coated with monoclonal anti-ASP antibody and blocked with bovine serum albumin (BSA). PEG precipitated plasma samples and in-house plasma controls were diluted 1:40 in phosphate-buffered saline (PBS) and added to the plate. ASP standard was also added using a concentration curve ranging from 0.156 to 10 ng/ml. After incubation, the plates were washed and incubated with rabbit polyclonal anti-ASP antibody. Subsequently, goat anti-rabbit IgG conjugated to horseradish peroxidase (Sigma-Aldrich) was added. The color reaction was initiated by the addition of ophenylenediaminedihydrochloride (Sigma-Aldrich) and was stopped using 1.25 M sulphuric acid. Absorbance was read at 490 nm. The intra-assay (within run) variation was 3.9%. IL-6, and MCP-1 were measured in plasma using DuoSet ELISA development kits from R&D Systems (Abingdon, UK). The intra-assay variation of IL-6 was 2.2% and MCP-1 was 2.8%.

### Statistical Analysis

The paired *t*-test was used to compare pre-post within group changes for clinical, hormonal and metabolic variables. The Wilcoxon signed rank test was applied to biochemical data that violated the assumptions of a normal distribution assessed by the Kolmogorov-Smirnov test. Between-group comparisons of percent changes of each parameters before and after treatment were performed using independent samples *t*-test. For all analysis, a two-tailed *P* ≤ 0.05 was considered to indicate statistical significance. Correlations of selected parameters were analyzed by using Pearson correlation coefficient and linear regression (forward stepwise regression). Statistical analysis was performed using SPSS for Windows NT, version 19.0 (SPSS Inc., Chicago, IL). Data are reported as mean ± SEM.

## Results

The mean age of women was 27.7 ± 1.4 years (atorvastatin group 26.6 ± 1.2 vs. placebo group 28.8 ± 1.8; *p* = 0.44). The BMI were comparable in both atorvastatin and placebo groups (33.20 ± 1.4 vs. 33.92 ± 1.4 kg/m^2^, *p* = 0.62) (17). There were no significant differences in baseline parameters between the two groups. After 12 weeks of atorvastatin therapy, there were significant reductions in total cholesterol (4.6 ± 0.2 vs. 3.4 ± 0.2 mmol/liter, *p* < 0.01), LDL-C (2.9 ± 0.2 vs. 1.8 ± 0.2 mmol/liter, *p* = 0.01), triglycerides (1.34 ± 0.08 vs. 1.08 ± 0.13 mmol/liter, *p* < 0.01), free androgen index (FAI) (13.4 ± 0.6 vs. 8.7 ± 0.4, *p* < 0.01), total testosterone (4.1 ± 0.2 vs. 2.9 ± 0.1 nmol/liter, *p* < 0.01), hs-CRP (4.9 ± 1.4 vs. 3.4 ± 1.1 mg/liter, *p* ≤ 0.04) and insulin resistance (as measured by homeostasis model assessment for insulin resistance, HOMA-IR) (3.3 ± 0.4 vs. 2.7 ± 0.4) along with significant increase in sex hormone binding globulin (SHBG) (31.1 ± 1.0 vs. 35.3 ± 1.2 nmol/liter, *p* < 0.01). There were no significant changes in any of these parameters in the placebo group (17). There was a positive correlation between reduction in HOMA-IR with improvements in FAI (*r*^2^ = 0.56; *p* < 0.04) and triglycerides (*r*^2^ = 0.68; *p* < 0.01) in the atorvastatin group (17).

The baseline ASP, IL-6, and MCP-1 were comparable in both groups (Table 1). There were significant reductions in ASP (156.7 ± 10.2 vs. 124.4 ± 9.8 ng/ml; *p* < 0.01), IL-6 (1.48 ± 0.09 vs. 0.73 ± 0.10 pg/ml; *p* < 0.01) and MCP-1 levels (30.4 ± 1.2 vs. 23.0 ± 3.5 pg/ml *p* < 0.02) after 12 weeks of atorvastatin (Table 1). The decrease in ASP (124.4 ± 14.8 vs. 119.5 ± 23.8 ng/ml *p* = 0.41), IL-6 (0.73 ± 0.34 vs. 0.74 ± 0.21 pg/ml *p* = 0.91), and MCP-1 (23.0 ± 4.5 vs. 23.9 ± 6.2 pg/ml *p* = 0.63) levels were maintained following 12 weeks of metformin treatment. There were no significant changes in ASP, IL-6, and MCP-1 levels in the placebo group.

**Table 1:** Comparison of inflammatory and adipose tissue dysfunction markers at baseline, 12 weeks of atorvastatin or placebo followed by 12 weeks of metformin.

**Variable**	**Atorvastatin pre-treatment group (*****n*** **=** **19)**							**Placebo pre-treatment group (*****n*** **=** **18)**						
	**Baseline (V1)**	**12 weeks (V2) (Atorvastatin 20 mg daily)**	**24 weeks (V3) (Metformin 1.5 gm daily)**	**V1/V2 (*p*-value)**	**%change V1–V2**	**V2/V3 (*p*-value)**	**V1/ V3 (*p*-value)**	**Baseline (V1)**	**12 weeks (V2) (Placebo)**	**24 weeks (V3) (Metformin 1.5 gm daily)**	**V1/V2 (*p*-value)**	**%change V1–V2**	**V2/V3 (*p*-value)**	**V1/V3 (*p*-value)**
ASP (ng/ml)	156.7 ± 16.2	124.4 ± 14.8	119.5 ± 23.8	<0.01	−20.0 ± 1.5 ^*^P < 0.01	0.41	<0.01	153.7 ± 20.1	135.6 ± 23.4	158.1 ± 32.8	0.37	−11.7 ± 3.2	0.16	0.52
IL-6 (pg/ml)	1.48 ± 0.29	0.73 ± 0.34	0.74 ± 0.21	0.01	−50.7 ± 3.9 ^*^P = 0.02	0.91	0.02	1.42 ± 0.28	1.21 ± 0.82	1.32 ± 0.25	0.54	−14.8 ± 5.2	0.86	0.73
MCP-1 (pg/ml)	30.4 ± 4.2	23.0 ± 4.5	23.9 ± 6.2	0.02	24.3 ± 2.2 ^*^P = 0.04	0.63	0.04	29.7 ± 4.9	27.1 ± 9.2	30.1 ± 4.6	0.30	−8.2 ± 1.9	0.49	0.97

*Atorvastatin pre-treatment group, Atorvastatin for 12 weeks followed by Metformin for 12 weeks; Placebo pre-treatment group, Placebo for 12 weeks followed by Metformin for 12 weeks; V1, Baseline; V2, 12 weeks from baseline on either atorvastatin or placebo; V3, 24 weeks from baseline (12 weeks from visit 2 on Metformin 1.5 g daily). Comparison between V1, V2, V3 in each group done using paired t-test*.

* *P, P-value for percentage difference between both group using unpaired t-test. Data are presented as mean ± SEM. All serum results are obtained from fasting variables*.

There were significant associations of ASP, IL-6, and MCP-1 with hs-CRP and testosterone levels. There were also substantial correlations of ASP and MCP-1 with improvements in insulin resistance (HOMA-IR); however, only ASP was shown to have positive correlation with significant improvements in triglycerides (Table 2).

**Table 2:** Correlation coefficients between changes in ASP, IL-6, and MCP-1 and changes in waist circumference, cholesterol, testosterone, hs-CRP, and HOMA after atorvastatin treatment.

***N* = 19**	**Parameters**	**Correlation coefficient**	***P*-value**
**Δ** **ASP**			
	Δ Weight	0.13	0.59
	Δ Waist circumference	0.12	0.78
	Δ Total cholesterol	0.24	0.66
	Δ Triglycerides	0.32	0.04
	Δ Testosterone	0.66	<0.01
	Δ hs-CRP	0.52	0.01
	Δ HOMA-IR	0.59	<0.01
**Δ** **IL-6**			
	Δ Weight	0.12	0.96
	Δ Waist circumference	0.16	0.90
	Δ Total cholesterol	0.22	0.52
	Δ Triglycerides	0.12	0.33
	Δ Testosterone	0.49	0.01
	Δ hs-CRP	0.57	<0.01
	Δ HOMA-IR	0.19	0.27
**Δ** **MCP-1**			
	Δ Weight	0.20	0.34
	Δ Waist circumference	0.10	0.95
	Δ Total cholesterol	0.11	0.82
	Δ Triglycerides	0.22	0.79
	Δ Testosterone	0.41	0.01
	Δ hs-CRP	0.44	<0.01
	Δ HOMA-IR	0.36	0.02

On multiple linear stepwise regression analysis the changes in ASP, IL-6, and MCP-1 accounted for 52% of the variance in CRP and 46% of testosterone levels but only ASP and MCP-1 accounted for 40% of the variance in HOMA-IR (Table 3).

**Table 3:** Multiple linear stepwise regression model identifying independent predictors of changes in testosterone, hs-CRP, and HOMA-IR.

**Dependent variable**	**Δ Testosterone**	**Δ hs-CRP**	**Δ HOMA-IR**
*R*^2^ (*p*-value)	0.46 (*p* = 0.01)	0.52 (*p* < 0.01)	0.40 (*p* = 0.03)
Independent variables	Δ ASP (*p* < 0.01) Δ IL-6 (*p* = 0.001) Δ MCP-1(*p* = 0.02)	Δ ASP (*p* = 0.01) Δ IL-6 (*p* < 0.01) Δ MCP-1 (*p* = 0.04)	Δ ASP (*p* < 0.01) Δ MCP-1 (*p* = 0.015)

*For dependent variables, Δ Testosterone, Δ hs-CRP, and Δ HOMA-IR were used. Independent variable included Δ ASP, Δ IL-6, and Δ MCP-1*.

## Discussion

This *post-hoc* analysis revealed that 12 weeks of atorvastatin treatment significantly decreased markers of adipose tissue dysfunction and inflammation as determined by ASP, IL-6, and MCP-1 in younger, overweight obese women with PCOS. These improvements were that are related significantly with substantial improvements in HOMA-IR, testosterone and hs-CRP levels.

In this study atorvastatin treatment improved ASP sensitivity in PCOS women with hyperandrogenimia) as suggested by significant reductions in ASP levels. This suggests a potentially novel mechanism for the improved insulin sensitivity reflected in the substantial decrease in HOMA-IR with associated improvements in androgen levels and inflammatory markers. In addition, reduction in ASP levels after by atorvastatin treatment was associated (although not necessarily causal) with significant improvement in dyslipidaemia in these women as suggested by enhanced triglycerides clearance and reduced cholesterol levels.

The reduction in ASP after 12 weeks of atorvastatin treatment was associated with substantial improvements in biochemical hyperandrogenaemia—the most distinctive feature of PCOS. Additionally, low levels of SHBG also contribute to high free testosterone levels by reducing binding with testosterone (20). Several studies have reported that insulin resistance with compensatory hyperinsulinaemia is the primary underlying cause for hyperandrogenaemia in women with PCOS. hyperandrogenaemia is induced by both decreased hepatic production of SHBG levels and increased production of androgens via ovaries and adrenal glands (21). This is evident further from intervention trials improving insulin resistance in PCOS women using different therapies e.g., weight loss, metformin, D-*chiro*-inositol and peroxisome proliferator-activated receptor gamma (PPARɤ)-agonists demonstrating reduced androgen levels with improved exaggerated androgenic response to ACTH, or LH stimulation tests (22–24). However, the underlying mechanism related to improved hyperandrogenaemia due to reduced insulin resistance has not been explained completely. Our findings suggest that an improved ASP resistant state and related intracellular pathways lead to improved insulin sensitivity that subsequently reduce androgen levels. Our study suggests the potential for novel treatment targets related to modifying adipose tissue dysfunction for PCOS women in the future.

Women with PCOS have elevated levels of hs-CRP that is an independent risk marker of early cardiovascular disease (25). There was 25% reduction in hs-CRP levels after atorvastatin treatment in the present investigation. These changes were was also positively correlated to ASP levels suggesting improvements in underlying inflammatory environment coincided with improved ASP sensitivity, although again, it is not possible to state whether one directly impacted on the other. However, these finding are consistent with previous studies aiming at improving cardiovascular risk in women with PCOS using several interventions including weight loss, metformin and PPARɤ (26, 27).

The changes in ASP were not correlated with changes in changes in total cholesterol and triglyceride suggesting that the effects of atorvastatin in these inflammatory markers are independent of its plasma lipid lowering effects. There is increasing body of evidence that there are beneficial effects of statin other than lipid lowering, or “pleotropic effects” including improvement in endothelial dysfunction, increase nitric oxide availability, antioxidant properties, inhibition of inflammatory responses, and stabilization of atherosclerotic plaques (28). These pleitropic effects could also be partly mediated through this effect on ASP resistance.

ASP levels have been shown to be increased in patients with obesity (4). In the current study both the groups are matched for body mass and there were no significant changes in weight before and after treatment with atorvastatin. This suggests that the effect of atorvastatin in ASP levels in women with PCOS is independent of changes in obesity status. This effect needs to be evaluated with normal weight or less overweight women with PCOS.

In women with PCOS there is a significant increase in proinflammatory cytokines especially IL-6 and MCP-1. This again appears to be independent of body mass index (29, 30). IL-6 is a potent inducer of hepatic CRP and has been implicated in cardiovascular atherosclerotic risk, dyslipidemia and hypertension (31). In this study there was a 50% reduction in IL-6 after 12 weeks of atorvastatin that correlated positively with reduction in CRP. The reduction in IL-6 appeared to be independent of reduction in total cholesterol and triglycerides with atorvastatin. Furthermore, the reduction in IL-6 was not related to changes in insulin resistance, suggesting reduction of ASP by atorvastatin as the underlying mechanism for reducing inflammatory markers.

It has been suggested that increased MCP-1 levels may induce insulin resistance (32) which are increased in women with PCOS compared with weight-matched controls (30, 33, 34). In this study, changes in MCP-1 levels correlated significantly with changes in ASP levels and independently predicted changes in HOMA-IR after atorvastatin therapy. The reduction in pro-inflammatory cytokines and subsequent insulin resistance could be due to improvement in underlying ASP resistance.

The reduction in ASP, IL-6 and MCP-1 was maintained after 12 weeks of metformin subsequent to atorvastatin therapy, suggesting metformin have an effect in continued suppression of these markers. Statins have also been shown to rapidly activate AMP-activated protein kinase (AMPK), a protein kinase that modulates metabolic homeostasis and energy balance in individual cells and multiple organs, both *in-vivo* and *in-vitro* (35). The actions of metformin also appear to be mediated by AMPK activation (36) suggesting that this pathway is independent of the ASP modulated effects.

In conclusion, 12 weeks of atorvastatin led to a significant reduction the adipose tissue dysfunction marker ASP and in adipocyte inflammation markers (IL-6 and MCP-1) in women with PCOS. The changes in ASP levels after atorvastatin treatment independently predicted changes in insulin resistance, androgens, and inflammatory markers. Our findings suggest that the effects of atorvastatin might be partly mediated through reducing ASP in women with PCOS.

## Data Availability

All datasets generated for this study are included in the manuscript and/or the supplementary files.

## Ethics Statement

All patients gave their written informed consent and the study was approved by the South Humber Research Ethics committee.

## Author Contributions

TS, JPH, ZJ, SC, A-MC, PP, AS, KC, and SLA were involved in the study design, acquisition of data, analysis and interpretation of data, and paper drafting. All authors read and approved the final manuscript.

### Conflict of Interest Statement

The authors declare that the research was conducted in the absence of any commercial or financial relationships that could be construed as a potential conflict of interest.
